# Electrochemical Microneedles for Real-Time Monitoring in Interstitial Fluid: Emerging Technologies and Future Directions

**DOI:** 10.3390/bios15060380

**Published:** 2025-06-12

**Authors:** Suhyeon Cha, Min Yu Choi, Min Jung Kim, Sang Baek Sim, Izzati Haizan, Jin-Ha Choi

**Affiliations:** School of Chemical Engineering, Jeonbuk National University, 567 Baekje-daero, Deokjin-gu, Jeonju-si 54896, Jeonbuk State, Republic of Korea; geth911@jbnu.ac.kr (S.C.); minyu5089@jbnu.ac.kr (M.Y.C.); minjeong413@jbnu.ac.kr (M.J.K.); harry1313@jbnu.ac.kr (S.B.S.); izzatihaizan22@jbnu.ac.kr (I.H.)

**Keywords:** electrochemical microneedle (MN), interstitial fluid (ISF), real-time monitoring, electrochemical detection, wearable sensor

## Abstract

Conventional blood-based detection methods for biomarkers and analytes face significant limitations, including complex processing, variability in blood components, and the inability to provide continuous monitoring. These challenges hinder the early diagnosis and effective management of various health conditions. Electrochemical microneedles (MNs) have emerged as a minimally invasive and highly efficient platform to overcome these barriers, enabling continuous molecular monitoring by directly accessing the interstitial fluid. Electrochemical MNs offer several advantages, including reduced patient discomfort, real-time data acquisition, enhanced specificity, and potential applications in wearable, long-term monitoring. In this review, we first analyze material selection and fabrication techniques to optimize sensor performance, stability, and biocompatibility. We then examine diverse detection strategies utilized in electrochemical MNs, including enzyme-based, aptamer-based, and antibody-based sensing mechanisms, each offering unique benefits in sensitivity and selectivity. Finally, we highlight the integration of electrochemical MN technology with multi-target detection, AI-driven analytics, and theragnostic capabilities. This convergence offers strong potential for smart healthcare and precision medicine. Through these technological innovations, electrochemical MNs are expected to play an important role in advancing continuous, noninvasive health monitoring and personalized medical care.

## 1. Introduction

The development of real-time monitoring sensors for in vivo analytes, such as biomarkers and drugs, is crucial for disease diagnosis and management, as well as in personalized medicine [1,2]. Biomarkers refer to a wide range of measurable indicators that reflect physiological conditions or pathological processes that are present in biological systems such as cells, tissues, and body fluids [3]. Detection and monitoring of biomarkers, including proteins, nucleic acids, peptides, and lipid metabolites [4], are essential in early disease diagnosis, monitoring disease progression, and therapeutic interventions [5,6]. Biomarker monitoring is important in modern medicine, offering enhanced diagnostic precision and enabling timely interventions to improve patient outcomes. It also facilitates immediate clinical decisions through continuous evaluation of disease progression and treatment response [7]. Therapeutic drug monitoring (TDM) is also essential for personalized therapy, particularly for determining the appropriate dosage of drugs with a narrow therapeutic window, such as antibiotics, for effective treatment [8].

Conventionally, blood or serum has been regarded as the gold standard for detecting analytes such as biomarkers and drugs. However, it presents several limitations, including complex procedures that result in prolonged processing times [7], limited sensitivity and specificity, and patient discomfort [9], leading to major constraints, especially in urgent medical conditions or when real-time data monitoring is required. In this regard, minimally or noninvasively accessible bodily fluids, such as interstitial fluid (ISF), urine, and saliva, are being explored as alternatives to blood. Among them, ISF is a rich source of various biomarkers and analytes, providing clinically important information [10]. ISF occupies the space between tissue cells and blood vessels, filling the gap between the extracellular matrix and cells. Blood travels through the capillary wall via osmotic pressure and pressurized filtration, allowing moisture and various other molecules to diffuse into the intercellular space [11,12]. This process involves the diffusion of various solutes (amino acids, hormones, peptides, etc.) from blood to ISF, so the biomarker composition between the two fluids is similar except for the presence or absence of blood cells [12,13]. In fact, studies have shown that there is little difference in the types of biomarkers found in plasma and ISF and that for certain skin-related biomarkers, their concentrations are even higher in ISF than in plasma [10,12]. Although there may be a time lag due to the time required for biomarkers detected in blood to diffuse into ISF, the difference is not substantial and predictable. Indeed, a study that monitored both plasma and ISF glucose levels in subjects demonstrated that the average delay was only about 5 to 6 min [14]. These properties highlight ISF as an innovative fluid of interest for disease diagnosis, personalized medicine, and a wide range of medical and biomedical applications [15].

MNs are generally designed to have a length ranging from 500 μm to 2000 μm, ensuring effective access to the ISF of the skin [16,17]. Due to these structural characteristics, MNs do not reach the nerve endings and capillary layers, thus enabling biomarker detection with minimal pain [18,19]. In addition, MNs have received significant attention in the biomedical field due to their low manufacturing cost and excellent suitability for mass production [20,21]. For biomarker detection, various techniques such as fluorescence, optical, and electrochemical methods have been integrated into the MN system [11]. In particular, electrochemical methods rely on the oxidation/reduction reaction of the target chemical into an electrical signal, enabling quantitative analysis [22]. This method exhibits a rapid response and sustained stability, making it appropriate for application in MN sensing systems [23], while its capability for on-site analysis provides the advantage of convenient measurements without the need for laboratory-based equipment [24]. Furthermore, it enables the acquisition of continuous physiological information, thereby maximizing the therapeutic effect, and has the potential to track the progress of disease in real time [10]. This approach, which combines the minimally invasive characteristics of the MN with the high analysis performance of the electrochemical sensor, can function as a new diagnostic platform that can more efficiently detect and manage health conditions by bridging the gap between existing analysis techniques and modern medical systems [25].

This review provides information on biocompatible electrochemical MN materials and precise manufacturing methods that increase electrochemical performance. It also highlights the role of different sensing strategies for improving efficient biomarker monitoring and further explores the potential of these strategies for multi-biomarker detection or combination with artificial intelligence (AI) systems. Such advancements could significantly increase the versatility of the technology, enabling more effective disease management and treatment through a multidisciplinary approach. Ultimately, we explore the impact of these advancements on the development of wearable biosensor technology and further explain electrochemical MNs as a next-generation smart biosensor platform.

## 2. Functional Materials for Electrochemical MNs

The performance of electrochemical MN sensors is largely dependent on the physical and chemical properties of the materials used in manufacturing. Since the electrical conductivity of electrochemical MN electrode materials directly affects sensor sensitivity and signal transmission efficiency, a careful deliberation of material selection upon application is imperative. Mechanical strength and biocompatibility are key factors that affect skin insertion and post-insertion safety. Therefore, materials that can effectively induce electrochemical reactions while minimizing skin tissue damage should be used. As a result, various functional materials such as highly conductive metals, conductive polymers, and carbon-based nanomaterials are being used in sensor design, and research is actively underway to precisely adjust sensor performance through structural and chemical deformation of these materials. In this section, specifically functional materials, including conductive metals, conductive polymers, 2D materials, and tunable materials, will be further discussed upon their application in electrochemical MN.

### 2.1. Conductive Metals

**Figure 1:**
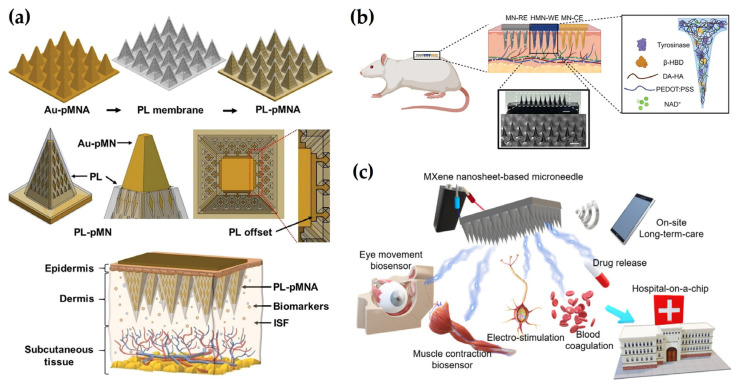
Materials integration in electrochemical-based MNs. (**a**) Schematic illustration of a PL-pMNA-based biosensing platform. Reprinted with permission from [37]. Copyright 2024, Wiley-VCH. (**b**) Schematic diagram of HMN-CKM device application on the back of a rat. Reprinted with permission from [65]. Copyright 2024, Wiley-VCH. (**c**) Graphical illustration of “hospital-on-a-chip” inspired by the MXene MN system. Reprinted with permission from [76]. Copyright 2021, American Chemical Society.

Conductive metals are expected to boost the conductivity of sensing materials as well as their signal amplification due to their outstanding electrical conductivity and wide specific surface area [26]. Within an electric field, free electrons pass through the lattice of metal nanomaterials and enable efficient current transfer, which improves the conductivity of the electrode surface and increases the detection sensitivity. Generally, highly conductive metals such as silver (Ag), gold (Au), platinum (Pt), and copper (Cu) are utilized as electrode materials [27]. Metals and metal alloys play a key role in the design of conductive MNs as they have excellent conductivity, ductility, and mechanical strength. In particular, metals and alloys such as stainless steel, titanium (Ti), and nickel (Ni) enable efficient signal transmission based on their high electrical conductivity, thus enabling the fabrication of durable MNs. These material properties are important factors in the development of electrochemical-based MN biosensors, improving signal processing efficiency and enabling precise detection of biomarkers [28]. Gold nanoparticles (AuNPs), a type of plasmonic nanoparticle, are widely used in sensors [29,30], cancer diagnosis, and treatment fields [31,32] owing to their high conductivity, large surface-to-volume ratio, and excellent biocompatibility. Through strong interactions with the thiol (-SH) group, AuNPs enable effective immobilization of biomolecules such as enzymes, antibodies, and nucleic acids to improve sensor selectivity and sensitivity. In addition, signal amplification is possible when AuNPs are used in electrode coating due to their excellent electrical conductivity and improved electron transfer speed. Due to these characteristics, AuNPs are particularly suitable for the development of electrochemical MN sensors [33,34,35]. For example, Jin et al. [36] designed a minimal invasive sensing system that monitors glucose levels in ISF of living mice using MN patches based on methacrylated hyaluronic acid (MeHA) deformed with Au/Cu_2_O nanospheres and screen-printed carbon electrodes (SPCE). The research group reported that doping Cu_2_O to Au improves conductivity and produces activated electrocatalysts for glucose oxidation, improving sensor detection performance. Similarly, Dervisevic et al. [37] developed a polymeric MN array (pMNA) platform coated with Au and protected by a 3D porous layer (PL membrane) for electrochemical detection of glucose and insulin in ISF. The Au layer serves as a conductive interface, enabling efficient electron transfer and providing a stable surface for immobilizing biosensing elements such as enzymes or aptamers. The PL membrane functions as a protective barrier that shields the MN surface from external mechanical and biochemical interference while maintaining the electroactive surface area of the Au coating. This unique configuration supports both high sensitivity and structural durability during skin insertion and biomarker detection (Figure 1a).

Zhang et al. [38] designed an MN-based glucose sensor to which AuNPs were introduced and compared its performance directly with a bulk gold electrode. The sensitivity of the bulk gold electrode immobilized with glucose oxidase (GOx) was 3.11 μA/mM, but the electrode to which AuNPs and overoxidized polypyrrole (OPPy) were applied showed a sensitivity of about 2.6 times to 8.09 μA/mM. This performance improvement is analyzed to have contributed to the large electroactive surface area of AuNPs and excellent electron transfer and hydrogen peroxide oxidation catalytic activity. In addition, the OPPy layer acted as an effective matrix for inducing uniform electrodeposition of AuNPs and increasing the immobilization efficiency of GOx, further improving the electrochemical response of the electrode. Downs et al. [39] developed an MN-based electrochemical aptamer sensor by embedding a 50 μm gold microwire into a commercially available stainless steel MN to form a miniaturized working electrode. The sensor specifically detected vancomycin within the clinical therapeutic range of 6 to 42 μM, demonstrating sufficient sensitivity for pharmacokinetic monitoring with an average accuracy of about 12%. Although the redox current was limited to the nanoampere scale due to its small electrode surface area, the authors noted that prior strategies, such as nanoporous gold structure and electrochemical roughness, can significantly amplify the current signal by improving the effective surface area by up to 100 times. Mechanically, the stainless steel MN showed excellent robustness, successfully penetrating porcine skin to a depth of about 1.3 mm without observable bending or damage during repeated insertions, thereby confirming its structural integrity for repeated transdermal application.

Meanwhile, platinum nanoparticles (PtNPs) are attracting considerable attention in application fields such as sensors [40,41] and electrocatalysts [42,43] due to their excellent biocompatibility, high surface-to-quality ratio, small size, and strong reactivity [44]. Specifically in electrochemical analysis, materials with excellent electrocatalytic activity, ease of synthesis, and stability in both acidic and alkaline electrolytes are preferred [45]. For instance, PtNPs promote catalytic oxidation of electroactive molecules such as dopamine (DA) by improving electron tunneling efficiency at electrode–reactor interfaces, which later increases catalytic activity at higher loading levels [46,47,48]. Furthermore, PtNPs can be synthesized through various technologies, including chemical reduction, metal-vapor synthesis, and electrochemical or photochemical deposition. These synthesis methods specifically have a significant impact on chemical inactivity and stability, with low background current as well as high catalyst and sensing capabilities of PtNPs [49]. For example, platinum black (PtB), known for its high surface area, excellent catalytic activity, and biocompatibility, is widely used for biosensor and electrode modification. PtB plays an important role in improving the electrical properties of electrodes. GhavamiNejad et al. [50] synthesized PtNPs and silver nanoparticles (AgNPs) in 3D porous hydrogels to develop inefficient electrochemical glucose sensors. Glucose was detected through the electrocatalytic dehydrogenation process, in which hydrogen atoms of the glucose C-1 bond chemically interact with the Pt and Au surfaces, resulting in oxidation to gluconolactone. This process enables the current generation even without oxygen and provides a stable sensing platform. In other work, Ming et al. [51] proposed a minimally invasive and continuous monitoring solution in the form of a portable pH MN sensor. The pH MN sensor was electrochemically modified to PtB and AuNPs to act as polyaniline (PANI) and increase sensitivity to hydrogen ions.

### 2.2. Conductive Polymers

Conductive polymers are electrically activated polymers, such as metal conductors, that exhibit conductivity through conjugated π electronic systems. The structure of these macromolecules consists of single and double bonds that are alternately arranged with delocalized electrons acting as charge carriers to exhibit electrical properties. Typical conductive polymers include PANI, poly(3,4-ethyleneedioxythiophene) (PEDOT), poly(p-phenylene), and polypyrrole (PPY). These polymers have adjustable electrical properties, excellent biocompatibility, and solution processability, making them highly advantageous for applications in electrochemical sensors, especially electrochemical MNs [27,52].

Among conductive polymers, PANI has a high specific capacity through multiple redox reactions as well as excellent electronic properties and thermal stability due to protonation. In addition, PANI is relatively easy and inexpensive to synthesize, attracting attention in various research and industrial fields [53,54]. As such, Mugo et al. [55,56] presented a flexible double-target electrochemical sensor capable of simultaneously sensing pH and cortisol. After coating the MN with PANI, the electron transfer resistance (Rct) of the electrode decreased from 3573 Ω to 2309 Ω, and the electroactive surface area increased about 13 times from 0.010 mm^2^ to 0.13 mm^2^, greatly improving conductivity and reactivity. This sensor reacted linearly in the pH range between 3 and 9. In another study, Liu et al. [57] designed MNs for monitoring uric acid (UA), active oxygen species, and pH in joints affected by gout. Correspondingly, PANI serves as a key component in the pH-sensing module of electrochemical MN sensors, functioning as a conductive and ion-selective polymer. Its integration significantly enhances the stability and reproducibility of electrochemical signals by facilitating selective proton detection while maintaining efficient electron transfer.

Additionally, PEDOT is a conductive polymer film with excellent stability, electrocatalytic activity, and high electron transfer rates [58]. In general, PEDOT is commonly applied as a conductor for electromagnetic wave absorption [59], energy storage devices [60], solar cells [61], and sensors [62]. The integration of polystyrene sulfonate (PSS) into PEDOT (PEDOT: PSS) results in a conductive polymer with high electrical conductivity, low oxidation potential, anti-contamination properties, and excellent thermal stability. PEDOT: PSS is often utilized for biomarker detection in combination with electrochemical sensing systems [63,64]. However, due to its low mechanical strength and brittleness, it is blended with other materials for use in MNs. Ausri et al. [65] designed a pH-monitoring MN consisting of a swellable DA-conjugated HA hydrogel embedded with PEDOT: PSS to improve the electrochemical sensing performance. This conductive hydrogel MN is capable of in vivo measurements with a 93% accuracy compared to a conventional pH probe meter (Figure 1b). Additionally, Odinotski et al. [66] developed a skin-compatible hydrogel MN device capable of continuously monitoring ketone bodies. In their work, PEDOT: PSS was introduced into a polyacrylamide-based conductive hydrogel to form an MN array capable of pH detection. The PEDOT:PSS complex hydrogel shows sufficient mechanical strength upon skin penetration and maintains its electrochemical function even when repeatedly bent and applied in vivo. Although no clear tensile strength values are presented, experimental evidence from insertion tests and strain stability suggests good mechanical robustness and flexibility. It maintains an electrical conductivity of about 10^−2^ S/cm, and shows a high sensitivity of 63.8 ± 1.2 mV/pH in the pH range of 4.0 to 8.0.

### 2.3. 2D Materials

Two-dimensional materials such as graphene, transition metal chalcogen compounds (TMDCs), and MXene can be adjusted in thickness from several nanometers to several centimeters [67,68]. In particular, the wide surface area and planar structure of 2D materials provide high binding capacity, excellent conductivity, and low signal-to-noise ratio (S/N ratio) with target molecules. These characteristics are essential in various applications such as electrochemical signal detection, bio-signal monitoring, and environmental sensing, as they greatly improve the system’s sensing performance. In addition, 2D materials have high transparency, flexibility, elasticity, biocompatibility, and selectivity depending on the thickness of the atomic layer. For example, the excellent conductivity of 2D materials such as graphene and MXene enables their application to detect various external substances. Moreover, their high sensitivity and stability make them highly promising for employment in the development of high-performance sensor platforms [67,69]. In brief, 2D materials can be optimized by sensor type and conversion mechanisms, offering better performance than existing bulk materials. Due to these advantages, 2D materials are gaining increasing attention as key components in next-generation electrochemical sensor technologies [69].

Graphene is a 2D carbon material with a wide specific surface area, excellent electrical conductivity, electron mobility, and mechanical strength, which is ideal for sensor electrodes and target molecule detection [70]. Unlike graphene, graphene oxide (GO) contains a large amount of oxygen-containing action groups (hydroxyl, carbonyl, epoxy, and carboxyl), imparting excellent hydrophilic properties and dispersibility in solution [71]. Furthermore, these functional groups facilitate interaction with various biomolecules and are suitable for biosensor development. In addition, GO can also be used in its flexible and transparent nanosheet form while maintaining its electronic, electrochemical, mechanical, and thermal properties. Among graphene derivatives, reduced graphene oxide (rGO) is a form of recovery product with electrical conductivity close to that of graphene, produced by removing GO oxygenators via chemical, electrochemical, high-temperature annealing, and ultraviolet irradiation. Compared to GO, rGO has higher electrical, optical, and mechanical properties, whilst also exhibiting thermal stability [71] similar to graphene. Additionally, its structural flexibility and ease of functionalization further support its integration into electrochemical MN platforms for biosensing applications [72]. For instance, Sharifuzzaman et al. [73] proposed an MN sensor consisting of cocktail M2, nicotinamide adenine dinucleotide (NAD^+^), NAD^+^-dependent glucose dehydrogenase, poly-l-lysine hydrobromide, and glutaraldehyde layer. In their study, rGO facilitated the cocktail absorption through the π-π interaction, resulting in improved electrical conductivity and sensor performance. Panicker et al. [74] developed a graphene-Ag-chitosan nanocomposite-based wearable electrochemical MN sensor for monitoring of serotonin in ISF. The developed sensors were able to detect 5-HT targets within a linear range of 3 to 21 μM owing to the fast electron transfer, excellent biocompatibility, and thermal conductivity of rGO.

MXene, a novel class of 2D materials composed of transition metal carbides, nitrides, and carbonitrides, possesses a layered structure exhibiting high electrical conductivity and excellent redox properties. The general chemical formula for MXene is expressed as M*_n_*_+_X*_n_*T_x_ (*n* = 1 to 3), where M represents transition metals such as Ti, Ta, molybdenum (Mo), hafnium (Hf), and zirconium (Zr). X denotes carbon (C) or nitrogen (N), while T means hydroxyl (OH), fluorine (F), chlorine (Cl), and oxygen (O). MXene is highly hydrophilic and can be easily combined with various functional substances due to its abundant surface action groups. The wide specific surface area and thin flake form provide excellent reactivity and high signal processing, and the high negative zeta potential allows for low detection limit (LOD) and wide sensing ranges [75]. Yang et al. [76] fabricated an MXene nanosheet-based MN for monitoring muscle contraction and electrical stimulation therapy and found that the MXene nanosheet-based MN is highly conductive, stable, and biocompatible (Figure 1c). In a different study, Zhang et al. [77] developed an electrochemical MN sensor based on MXene for rapid and accurate glucose detection in ISF. To address the intrinsic restacking tendency of MXene and improve its electrochemical performance, cerium oxide (CeO_2_) and multi-walled carbon nanotubes (MWCNTs) were incorporated into the electrode design. The resulting composite sensor demonstrated high sensitivity, a broad detection range, and fast response time, highlighting its potential for noninvasive and continuous glucose monitoring.

### 2.4. Tunable Materials

Quantum dots (QDs) and metal-organic frameworks (MOFs) are tunable materials that offer adjustable size and structural versatility, respectively, making them suitable for electrochemical MN biosensing applications. Briefly, QDs are nanocrystals known for their distinct optical and electronic properties, allowing precise modulation of signal transduction and enhanced sensitivity through size-dependent characteristics [78]. In the field of electrochemical detection, QDs are commonly employed in photoelectrochemical (PEC) sensor platforms as photosensitizers to enhance the photoelectric response of opto-electrodes [79]. The PEC system operates on the principle of light-to-electron conversion, enabling high sensitivity, rapid response, and low-cost detection [80]. Within the MN field, the integration of QDs offers enhanced signal detection while reducing patient discomfort, due to their ability to emit fluorescence upon exposure to near-infrared (NIR) light [78]. Nonetheless, within the scope of this review, which focuses on electrochemical MNs, we highlight the application of QDs in MN-based fluorescence sensing as a promising direction. For instance, Li et al. [81] developed a c-GelMA-MeHA double-network hydrogel MN-based assay incorporating carbon QDs (CQDs) for the detection of Cu^2+^ in ISF of breast cancer patients. Upon insertion into porcine cadaver skin, Cu^+^ ions mediated fluorescence quenching of CQDs, enabling sensitive detection.

MOFs, on the other hand, are porous crystalline materials composed of metal ions or clusters coordinated with organic ligands. They offer a high degree of tunability in surface chemistry, pore size, and catalytic functionality [26]. In electrochemical sensing, the large surface area of MOFs facilitates enhanced analyte adsorption and promotes effective interaction and mass transfer between analytes and the electrode surface, ultimately improving signal response and sensitivity. Furthermore, due to their inherent catalytic properties, MOFs can serve as active catalytic electrode materials [82]. Despite their potential, the integration of MOFs into electrochemical MN platforms remains underexplored and represents a critical gap in the field. Nonetheless, relevant examples exist in fluorescence-based MN sensors. For example, Liu and co-workers [83] developed a wearable MN patch integrated with europium-based MOFs (Eu-MOFs) for cortisol monitoring in ISF. By leveraging the spin–orbit coupling, effective antenna ligands, and strong magnetic moments from the 4f^n^ electron configuration of Eu^3+^, the system exhibited high cortisol selectivity and strong molecular interactions, leading to fluorescence quenching that enabled improved quantitative analysis and visible stress monitoring [84,85,86]. Moving forward, the exploration and integration of functional nanomaterials such as QDs and MOFs into electrochemical MNs are expected to advance the development of continuous, noninvasive sensing systems for biomedical applications.

## 3. Fabrication Strategies for Electrochemical MNs

Electrochemical MNs require not only sufficient penetration ability but also adequate mechanical strength [87] and precise shape retention to ensure efficient and reliable skin insertion [88]. To ensure stable insertion into biological tissue without structural damage, key structural parameters such as MN length, tip geometry, and density need to be meticulously designed [89]. This is because the outlined factors directly impact the overall sensor performance and measurement reliability. To meet these requirements, various high-precision microfabrication techniques are employed. Ultimately, the precision and reproducibility of the fabrication process play a critical role in determining the consistency and accuracy of the resulting electrochemical signals [90]. A detailed list of Comparison of fabrication methods for electrochemical MN sensors is presented (Table 1).

### 3.1. Photolithography

Photolithography is a precise microfabrication technique that utilizes ultraviolet (UV) light or a laser to form microscale patterns on photosensitive materials (photoresists) [99]. This technique enables high-resolution transfer of desired microstructures onto the photoresist via a mask or reticle, followed by processes such as etching or replica molding to construct the MN structures with high precision [100]. With its ability to achieve resolution in the tens to hundreds of nanometers while maintaining high pattern fidelity, photolithography allows for the fabrication of complex MN architectures [101]. As the sensitivity and selectivity of electrochemical sensors are significantly influenced by the dimensional precision, surface characteristics, and spacing between electrodes, photolithography ensures these parameters can be controlled with high uniformity and reproducibility [102]. Thereby, photolithography contributes to the development of reliable monitoring platforms. Moreover, the photolithographic process is well-suited for wafer-scale batch manufacturing and automation, making it highly effective in biosensor-integrated electrochemical MN system development that requires consistent sensor performance and scalable fabrication [103]. Its compatibility with various conductive materials further enables the integration of multi-layer structures and precise electrode patterning, allowing flexible application in the development of advanced MN platforms.

**Figure 2:**
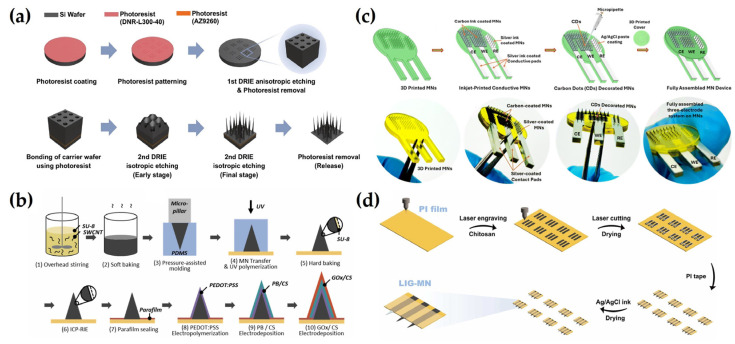
Fabrication methods for electrochemical-based MNs. (**a**) Schematic illustration of MN fabrication using subsequent photolithography and DRIE processes. Reprinted with permission from [91]. Copyright 2021, Springer Nature. (**b**) Schematic diagram of SU-8/SWCNT MN fabrication using casting and molding methods. Reprinted with permission from [93]. Copyright 2023, Elsevier. (**c**) Schematic of MN design using 3D printing and MN electrode fabrication using inkjet printing. Reprinted with permission from [95]. Copyright 2025, Royal Society of Chemistry. (**d**) Schematic diagram of LIG-MN patches using laser cutting technology. Reprinted with permission from [97]. Copyright 2024, Elsevier.

In a research study, Roh et al. [91] proposed a high-density out-of-plane MN array characterized by varying heights and cross-sectional shrapes using a single photolithography and two deep-reactive ion etching (DRIE). The fabrication approach allows precise control over MN height distribution and cross-sectional shapes through photomask design, and the entire process is conducted via dry etching, eliminating the need for wet etching steps while ensuring both simplicity and structural precision. The resulting MN arrays achieved a high density of up to 625 MNs/mm^2^ and a maximum aspect ratio of 25, demonstrating sharp and intricate needle structures. Notably, isotropic DRIE enabled the formation of ultra-sharp needle tips with a minimum diameter of approximately 145 nm, significantly reducing the insertion force and minimizing tissue damage during penetration. The fabricated MNs also exhibited excellent structural uniformity with height and width variations maintained within 1.2% and 1.4%, respectively. This study presents a robust and versatile fabrication strategy well-suited for the development of high-precision MN systems that meet diverse functional and structural requirements (Figure 2a). In another work, Dardano et al. [92] proposed a simplified fabrication method for a hollow MN, addressing the limitations of conventional multi-step processes by employing a single-step photolithographic approach. In this study, a mixture of polyethylene glycol diacrylate (PEGDA) and a commercial photoinitiator was used as a negative photoresist to simultaneously form both the outer structure and the internal cavity of the hollow MN, eliminating the need for etching steps. The study further demonstrated high skin permeability and strength of MNs by precisely controlling the height of the MN fabrication to 240 μm with an opening size of 75 μm by adjusting the time of UV exposures. Moreover, the same photolithographic process enabled the simultaneous fabrication of both the MNs and their supporting patch substrate, highlighting the potential for scalable manufacturing and the integration of hollow MNs into fully functional bio-devices.

### 3.2. Casting and Molding

Casting and molding is a representative technique to simply and precisely manufacture MNs, involving the fabrication of a highly precise master structure, which is then replicated using an elastomeric mold to produce the final MNs [104]. Initially, a master MN structure is fabricated using materials such as silicon wafers or metals, which serve as a template for subsequent replication [105]. A mold is then produced using an elastomer such as PDMS, which is renowned for its excellent elasticity, biocompatibility, and high-fidelity replication of microstructures, making it a standard material in MN manufacturing [106]. The mold cavities are subsequently filled with a polymer or hydrogel solution, which undergoes a curing process to form the final MNs [107]. This method supports the use of diverse materials and enables the integration of functional elements such as conductive polymers and nanomaterials, thereby facilitating the development of electrochemical MNs with sensing capabilities. Moreover, the high reproducibility and reusability of the mold contribute to cost-effectiveness and scalability, allowing for the mass production of MNs with consistent quality and reliable performance [108].

For instance, Kim et al. [93] introduced a casting and molding-based manufacturing technology known as the mold-and-place method, which uses a composite of photocurable polymer SU-8 and single-walled carbon nanotubes (SWCNTs) to make individually addressable electrochemical MN electrodes, to form an MN electrode system by partially metalizing the existing MN electrode. In this approach, the SU-8/SWCNT mixture was poured into a PDMS mold placed on a pre-patterned electrode substrate and cured under UV light to form an MN structure, and the concentration of SWCNT was demonstrated to be high in moldability and strength at 0.5 wt%. The process employs pressure-assisted transfer (PAT) molding, which enables precise replication and alignment of MNs onto both rigid and flexible substrates. To enhance electrochemical performance, the fabricated MNs undergo inductively coupled plasma reactive ion etching (ICP-RIE), which improves their surface characteristics and conductivity. This mold-and-place strategy offers a scalable and adaptable platform suitable for multiplexed sensing applications (Figure 2b). In a different study, Zhao et al. [94] developed a stretchable MN electrode array than conventional silicon-based MNs using hybrid fabrication methods that integrate laser micro processing, replica molding, microfabrication, and transfer printing. The core of the process is casting and molding, where PDMS molds are laser-ablated to form conical cavities that define the MN geometry. These molds are filled with polyimide and cured to form high-modulus MNs, after which Cr/Au metal layers are deposited onto the MNs and integrated onto an elastomeric substrate to complete the electrode array. The resulting MNs exhibit high mechanical strength, stretchability of up to 90%, and excellent electrical stability, demonstrating that the casting and molding process is an effective technique for fabricating high-performance bioelectronic interfaces.

### 3.3. 3D Printing

Three-dimensional printing has emerged as a precise and versatile fabrication method for MN production, allowing for the direct construction of microstructures without the need for molds. Photopolymerization-based techniques such as stereolithography (SLA), digital light processing (DLP), and two-photon polymerization (2PP) enable the high-resolution fabrication of fine needle geometries and complex structures [109]. The ease of design modification also makes this approach well-suited for rapid prototyping. Moreover, a wide range of biocompatible and conductive materials can be employed, facilitating the integration of electrochemical sensing functionalities into MNs [110]. As such, 3D printing offers key advantages, including high precision, design flexibility, and simplified manufacturing processes, positioning it as a promising strategy for the development of MN-based biosensors [111].

For example, Kadian et al. [95] fabricated a 3D-printed conductive MN array-based electrochemical point-of-care device for transdermal sensing of chlorpromazine. The MN array was made with Projection Micro Stereolithography (PμSL) technology using BIOS resin, of which the MN sequence was set to a height of 1200 μm and a basic diameter of 350 μm. Following fabrication, the MNs were modified through inkjet printing of conductive carbon and Ag inks to form a three-electrode electrochemical system, with carbon dots incorporated into the working electrode to enhance signal sensitivity. The developed MN array demonstrated an adequate strength for penetration into porcine skin, with minimal impact on the surrounding tissue, proving a minimally invasive MN array. This minimally invasive platform demonstrated high sensitivity, good linearity, and reliable performance in a skin-mimicking model, supporting its potential for wearable drug monitoring applications. These results highlight the effectiveness of 3D printing in producing high-performance, minimally invasive MN sensors, demonstrating strong analytical capabilities and practical potential for wearable drug monitoring (Figure 2c). In other work, Liu et al. [96] developed a wearable MN biosensing patch for continuous glucose monitoring using a 3D printing-based fabrication process. The MN array was fabricated by SLA 3D printing using transparent photosensitive resin, with a height of 800 μm, a bottom diameter of 400 μm, and a gap of 200 μm allowing precise configuration of sharp MN with high mechanical strength and reproducibility. After printing, the MN surface was coated with Au and Ag/AgCl layers to form a three-electrode electrochemical system. This 3D printing approach allowed for accurate control over MN geometry, ensured batch-to-batch consistency, and simplified the integration of sensor layers onto complex microstructures. The resulting platform demonstrated reliable performance in both simulated body fluids and diabetic mouse models, validating the feasibility of 3D-printed MNs for continuous, minimally invasive glucose sensing.

### 3.4. Laser Cutting

Laser cutting, on the other hand, is an advanced manufacturing technology that utilizes high-power lasers to precisely cut substrates and is an important process in the manufacture of MNs. This technique can be applied to a variety of materials, such as metals, polymers, and silicon, wherein it is especially effective in processing polymers and biomaterials to suitably produce medical usage MNs that customarily require biocompatibility [112]. One of the biggest advantages of laser cutting is its non-contact processing, which can accurately form fine structures without damaging the materials [113]. This allows for precise adjustment of the length, diameter, and arrangement of the MNs and provides a design that is optimized for the desired functionality and application [114]. Additional post-processing processes are rarely required, boosting the efficiency of the overall production process and maintaining consistent quality between products. In other words, this is a very important factor for biosensor applications that require MNs to maintain uniform penetration depth and structural stability. For this reason, laser cutting technology has been positioned as an important processing method in MN manufacturing, especially as it has the advantages of being high in speed with precise pattern definition [115]. Therefore, with continuous technology development, more sophisticated and efficient MN production will be expected.

As an example, Sun et al. [97] use flexible laser-guided graphene (LIG) and have fabricated multifunctional MN patches through a simple and fast laser-based fabrication process. A CO_2_ laser was employed to directly engrave conductive graphene electrodes onto polyimide (PI) films and to precisely define the MN structures, enabling rapid, scalable, and mold-free manufacturing. As PI material has been widely used as a substrate material due to its excellent mechanical strength and flexibility, the fabricated MNs exhibited sufficient mechanical strength for reliable skin penetration without structural damage, allowing effective access to ISF. This laser-assisted strategy also facilitated the seamless integration of electrochemical sensing capabilities. As a result, the LIG-MN patch demonstrated high sensitivity and selectivity for detecting glucose and vitamin C in ISF (Figure 2d). In a different study, Huang et al. [98] developed a multi-channel MN electrode system through a separated functionalization and assembly process to reduce crosstalk between electrodes, resulting in enhanced skin penetration and sensing selectivity. In brief, stainless steel sheet was laser-cut in a quick and uniform manner to fabricate three electrode substrates, which were then combined with 3D-printed resin to form an MN array. The length of the MN was about 800 μm and the diameter was about 200 μm, which was sufficient to achieve safe and stable skin penetration for ISF detection. The working electrodes were sequentially coated with Au, Pt, and glucose oxidase, while the reference electrode was coated with Au and Ag/AgCl ink. These MNs were rapidly fabricated, and the modular assembly method improved detection performance, demonstrating great potential for adaptation in various sensing platforms. Overall, laser cutting complements other MN fabrication techniques by offering rapid, precise, and scalable production, which is especially valuable for applications requiring high-throughput and reliable sensor performance.

## 4. Electrochemical Sensing Strategies in ISF Analysis

Electrochemical sensors enable detection of vital bio-signals and biomarkers in the body, facilitating disease management [116]. Electrochemical MN allows for more sensitive and accurate small molecule analysis by directly analyzing body fluids through MNs under the skin. This technology can be used to continuously monitor metabolic biomarkers [117], chronic illness biomarkers such as cancer [19], and drug concentrations after their administration [118,119]. Real-time data allows patients to respond quickly to changes in their health status and take immediate medical action.

In connection with this, the synergy between a sensor’s fundamental principles and its sensing mechanisms is a key part of this technology. Electrochemical MNs detect and analyze electrical signals generated by the reaction with target molecules, resulting in highly sensitive and accurate analysis results [120,121,122]. Understanding the various sensing mechanisms is essential for optimizing sensor performance, allowing them to be tailored to specific medical or experimental conditions [123]. It is also imperative to identify the latest technological advances and expand their applications in medical diagnosis and personal health monitoring. It is believed that the expansion of these technologies can contribute to improving patient care by accelerating early detection of diseases and treatment decisions. The development of optimized sensors would facilitate a more economical cost as well as accessibility of testing. Thus, understanding the various sensing mechanisms provides valuable resources for clinical applications and scientific research [124]. This is an important step in facilitating scientific and clinical advances, authorizing technological accessibility to a wide range of users. A detailed list of Comparative summary of receptor types used in electrochemical MN sensors is presented (Table 2).

### 4.1. Direct Label-Free Detection

Some biomarkers and drugs are electrochemically active substances, wherein they can undergo direct redox reactions at the electrode surface [137]. Direct detection methods utilizing these properties make electrochemical MNs advantageous due to their simple construction, low cost, and stable performance over the long term [138]. In addition, direct label-free detection is suitable for monitoring systems because it has low sensitivity to external environmental factors such as temperature and pH changes, ensuring a fast response speed and excellent reproducibility [139]. However, this sensing strategy suffers from intrinsic limitations in selectivity due to direct redox reactions occurring at the electrode interface, making it vulnerable to interference from compounds with electrochemical properties similar to those of the target biomarkers [140]. Furthermore, the non-specific accumulation of proteins and cellular residues under physiological conditions induces biofouling, which contaminates the electrode surface and degrades the signal [141]. To overcome this limitation, surface modification techniques aimed at enhancing the selectivity of the electrode, along with the incorporation of protective layers to block interfering substances, have emerged as important strategies.

**Figure 3:**
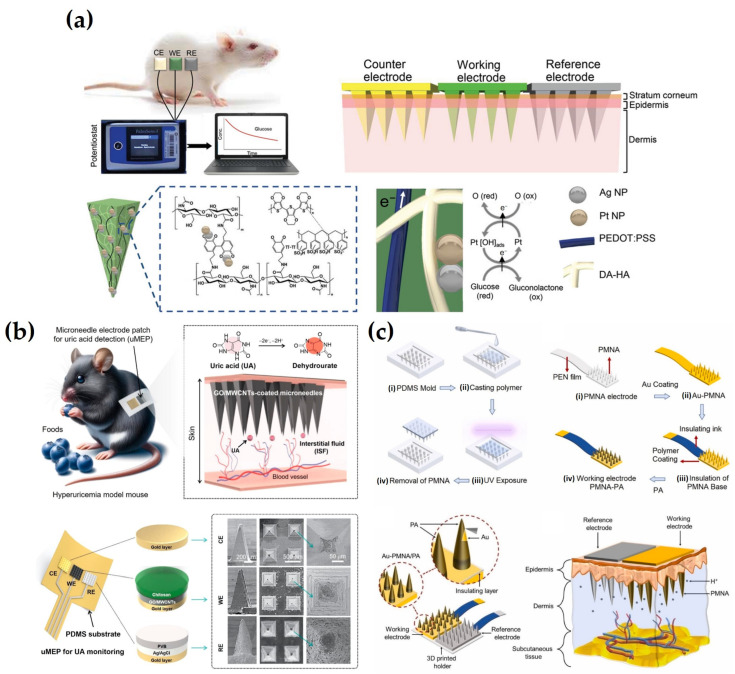
Direct detection in ISF using electrochemical MNs. (**a**) Schematic graphic of the HMN-CGM system for glucose monitoring using an NP-integrated HMN electrode. Reprinted with permission from [50]. Copyright 2022, Wiley-VCH. (**b**) Schematic illustration of uMEP for UA sensing in ISF via GO/MWCNT MNs. Reprinted with permission from [126]. Copyright 2024, American Chemical Society. (**c**) Schematic diagram of the fabrication and application of PMNA-based MN electrode patch enabling direct electrochemical sensing. Reprinted with permission from [142]. Copyright 2024, Elsevier.

As an example of this approach, Tortolini et al. [125] developed nanoporous gold (nPG)-based MN sensors capable of continuous monitoring of catecholamine-based hormones, including DA, epinephrine (EP), and norepinephrine (NEP). The nPG layer enhanced electrode properties by increasing the surface area, reducing electron transfer resistance, and minimizing nonspecific adsorption, thereby improving sensitivity and selectivity. These property improvements also contributed to overcoming interference from species with overlapping oxidation potentials, enabling clear peak separation and selective detection of catecholamines. Additionally, a LOD of 0.3 μM for NEP was achieved in gel skin models, suggesting stable and long-term performance under practical conditions. In a different study, Panicker et al. [74] developed an MN sensor based on an Ag/rGO/chitosan nanocomposite for continuous electrochemical monitoring of serotonin (5-hydroxytryptamine, 5-HT). Nanocomposite incorporation enhanced electrode conductivity, sensitivity, and stability. Additionally, a Nafion protective layer was applied to prevent the leaching of electrode components and to electrostatically block negatively charged interfering species such as ascorbic acid (AA), UA, and EP, thereby improving selectivity. This combination of surface modification and protective layering contributed to antifouling performance and signal stability during prolonged monitoring, with less than 8% signal variation over 3 h in artificial interstitial fluid (aISF). As a result, LOD of 5.3 μM in artificial aISF and 0.9 μM in gel skin models was achieved, confirming its potential for sensitive and reliable real-world monitoring. Ghavami Nejad et al. [50] fabricated a hydrogel-based MN continuous glucose meter (HMN-CGM) to enable glucose detection. To enhance electrode selectivity and conductivity, a conductive polymer, PEDOT: PSS, was incorporated into the DA-HA hydrogel to establish a stable electron transfer pathway, while PtNPs and AgNPs were simultaneously synthesized within the hydrogel to maximize catalytic activity and signal amplification. The resulting nanocomposite network enhanced charge transport and structural robustness, while the hydrogel served as a biocompatible membrane that protected against mechanical stress and biofouling during prolonged skin contact. This structure effectively suppressed signal distortion caused by interfering substances such as AA and UA, facilitating direct electrochemical detection of glucose. As a result, the current response increased linearly with glucose concentrations ranging from 0 to 35 mM in aISF, achieving a correlation coefficient (R^2^) of 0.99 and a detection limit of 0.9 mM. The sensor also maintained stable performance for 14 days when attached to the skin of STZ-induced diabetic rats, accurately tracking blood glucose fluctuations and showing a high correlation with conventional glucometer readings, thereby demonstrating the effectiveness of continuous monitoring (Figure 3a). To enable continuous electrochemical monitoring of UA, Zhao et al. [126] developed an MN electrode patch by modifying the electrode surface with a composite of GO and carboxylated MWCNTs, followed by the application of a 0.1 wt% chitosan coating. This surface functionalization improved conductivity, enhanced selective oxidation of UA via oxygen-containing functional groups, and electrically blocked negatively charged interferents such as AA. Additionally, the chitosan layer served as a biocompatible membrane that helped prevent nonspecific adsorption and surface fouling. In a skin-like gel environment, the sensor exhibited a linear response over 0–500 μM with a detection limit of 0.16 μM, while maintaining a strong correlation (R^2^ = 0.97) between UA concentrations in ISF and blood, demonstrating the feasibility of continuous monitoring (Figure 3b). Devisevic et al. [142] developed a system to continuously monitor the pH in ISF using a potential difference sensor based on a polymer MN array (PMNA). The electrode surface was modified with electrochemically polymerized PANI, enabling selective potential responses sensitive to proton (H^+^) concentration through conductivity changes associated with the emeraldine salt (ES) state. This system achieved precise pH detection over a range of 4.0 to 8.6 with a sensitivity of 62.9 mV/pH, exhibiting negligible potential shifts in the presence of interfering ions such as Na^+^, K^+^, Ca^2+^, and Mg^2+^. A highly linear potential response (R^2^ = 0.9992) was demonstrated in aISF, and accurate pH changes were detected upon application to mouse skin. These results confirm the bio-applicability of MN sensors based on pH-specific electrode modification and structural shielding (Figure 3c). Molinero-Fernandez et al. [143] fabricated an MN sensor for carbon dioxide (CO_2_) detection. The sensor integrated pH- and carbonate (CO_3_^2−^)-selective electrodes, each coated with an ion-selective membrane (ISM), to enable direct ion detection through potential differences. A functionalized multi-walled carbon nanotube (f-MWCNT) layer was introduced to enhance signal transmission efficiency, and a PU coating was applied to the reference electrode to improve stability against external interference. Inserted into the back skin of anesthetized mice, the sensors exhibited rapid responses within 5 s and enabled continuous monitoring of CO_2_ levels at 10 min intervals, outperforming conventional blood gas analyses. This approach constitutes a promising strategy for noninvasive respiratory monitoring with high selectivity and fast response. In conclusion, many studies have continuously attempted to improve the performance of direct detection methods by modifying the structure of electrode materials and controlling the physicochemical properties of the protective layer. Based on the literature, this approach complements the existing limitations and further improves the practicality of electrochemical MN sensors.

### 4.2. Enzyme-Based Detection

Enzyme-based biosensors have been conventionally utilized due to their high biocatalytic activity and specificity [144]. Enzymes are widely used as catalysts due to their strong reactivity, excellent selectivity, and substrate specificity, all of which contribute to the generation of stable and reproducible signals [145]. These properties allow enzyme-based sensors to detect a wide range of analytes. The enzyme-based sensors operate by electrochemically detecting redox reactions occurring at the electrode surface [146], where the enzyme reacts with a specific substrate to produce redox-active byproducts [147]. The electron flow resulting from the enzymatic reaction is detected by the sensor and analyzed in real time to accurately measure the biomarker concentration [148]. This allows for rapid and accurate diagnosis, offering significant potential in medical diagnostics and health monitoring applications [149]. Incorporating enzyme sensors into MNs would allow direct access to the ISF through the skin, allowing continuous monitoring of chemical changes in the body [150]. However, due to their high sensitivity to environmental factors such as temperature and pH, enzymes are prone to denaturation and loss of activity, and immobilization onto electrode surfaces without compromising catalytic function remains challenging [151]. Moreover, maintaining optimal conditions in real measurement environments is often difficult, making it challenging to ensure reproducibility and stability in complex biological matrices [152]. Thus, it is essential to develop strategies to ensure consistent sensor performance while preserving enzyme activity and enabling effective catalytic function.

**Figure 4:**
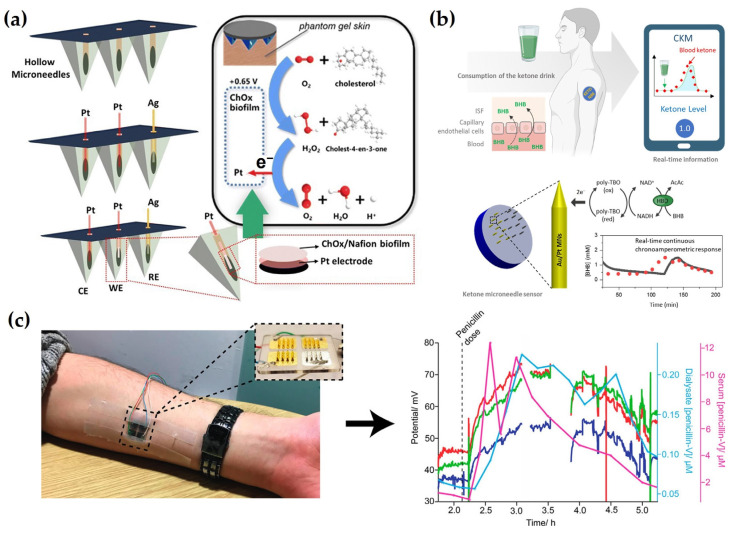
Enzyme-based detection in ISF using electrochemical MNs. (**a**) Schematic graphic of cholesterol detection using a ChOx/Nafion-functionalized Pt electrode integrated into a hollow MN array. Reprinted with permission from [128]. Copyright 2024, Royal Society of Chemistry. (**b**) Schematic illustration of BHB monitoring in ISF using an MN sensor with selective NADH oxidation and wireless readout. The black line in the chronoamperometric response graph represents sensor data, while red dots indicate finger-prick validation. Reprinted with permission from [153]. Copyright 2024, American Chemical Society. (**c**) Illustration of monitoring of β-lactam antibiotics using an MN sensor via β-lactamase-induced pH change. Graph shows the in vivo response of three β-lactamase microneedle biosensors (red, green, and dark blue) applied to a human subject, along with serum (pink) and dialysate (light blue) penicillin V concentrations over time. Reprinted with permission from [129]. Copyright 2019, American Chemical Society.

Ming et al. [127] designed an electrochemical MN sensor using AuNPs and PtNPs for glucose monitoring. The inclusion of AuNPs increased the surface area and enhanced electrical conductivity, while PtNPs immobilized on the MNs effectively detected hydrogen peroxide generated from the enzymatic reaction between GOx and glucose. A PU protective coating was subsequently applied to improve in vivo stability. After 7 days of storage in bovine serum, the signal degradation was significantly lower in the PU-coated sensor (13.13%) than in the uncoated sensor (45.06%), further confirming the protective role of the PU layer in preserving enzyme activity and stabilizing the sensor signal. This improvement in stability highlights the potential for in vivo applications of the sensor and supports its future use in minimally invasive continuous glucose monitoring systems. In a different study, Li et al. [128] developed a cholesterol detection sensor by integrating Pt and Ag electrodes into a pyramid-shaped MN with a microcavity structure. A mixture of cholesterol oxidase (ChOx), bovine serum albumin (BSA), and Nafion was drop-cast onto the Pt electrode for enzyme immobilization. The microcavity structure contributed to the signal sensitivity by protecting the enzyme activity, improving the reaction efficiency, and providing sufficient space for the interaction between cholesterol and the enzyme. BSA helped to preserve the structural integrity of the enzyme, while Nafion improved the enzyme retention and acted as a barrier to interferences. The sensor demonstrated long-term stability by retaining 86% of the initial response after four weeks of storage and showed high reproducibility with 2.05% relative standard deviation (RSD) among identically prepared sensors. In a skin-mimicking phantom gel, the sensor showed a linear response to cholesterol concentrations in the range of 2 to 10 mM with R^2^ = 0.9996, confirming excellent analytical precision and sensitivity. This study demonstrated the potential of the developed platform for noninvasive cholesterol monitoring (Figure 4a). For continuous monitoring of β-hydroxybutyrate (BHB), a major ketone body in blood, Moonla et al. [153] constructed an MN sensor. The MN surface was modified with poly(toluidine blue O) (poly-TBO) to selectively catalyze the oxidation of NADH at a low potential of +0.2 V whilst minimizing interference from other substances and enhancing catalytic efficiency. To promote efficient electron transfer and ensure stable immobilization of β-hydroxybutyrate dehydrogenase (HBD), a composite layer of carboxylated CNTs and chitosan was applied. Subsequently, the chitosan and PVC layers served as semipermeable and permselective outer coatings, effectively minimizing non-specific adsorption and biofouling on the electrode surface. After 15 days of storage at 4 °C, the sensor maintained 92% of its initial sensitivity and showed high reproducibility, with 2.08% RSD. In human trials, the sensor successfully detected changes in ISF BHB concentration following ketone beverage consumption and showed a strong correlation with blood BHB levels. These results validate the effectiveness of the sensor for in vivo monitoring and highlight its potential utility in managing metabolic disorders (Figure 4b). In a separate study, Gowers et al. [129] fabricated a potentiometric MN-based biosensor for monitoring β-lactam antibiotic concentrations. The sensor incorporated a pH-sensitive iridium oxide (IrOx) electrode and a hydrogel matrix embedded with β-lactamase enzyme. The hydrogel, composed of polyethyleneimine (PEI) and poly(ethylene glycol) diglycidyl ether (PEG-DE), provided a stable environment that preserved enzymatic activity and enhanced reaction efficiency. Additionally, a PEI outer coating was applied to prevent enzyme leaching and to improve mechanical stability. After two weeks of storage at −20 °C, the sensor maintained a sensitivity of 10.8 ± 0.35 mV/mM, demonstrating robust long-term stability and reproducibility. In human trials, the sensor successfully tracked changes in penicillin V concentration through the skin, generating signal patterns consistent with blood concentration and microdialysis measurements. These results highlight the clinical applicability of the developed sensor for TDM and its potential role in personalized antibiotic therapy (Figure 4c). These studies collectively highlight the performance of enzyme-based electrochemical MN sensors, which is critically dependent on enzyme activity preservation, long-term stability, and consistent reproducibility. The studies reviewed above explored various strategies to address these factors, including optimization of electrode structures, improvement of enzyme immobilization techniques, and tuning of protective layer properties. Continuous advances in these areas are expected to further expand the practical utility of enzyme-based MN sensors as noninvasive and high-precision biosensing platforms.

### 4.3. Aptamer-Based Detection

Aptamers are single-stranded oligonucleotides that specifically bind to proteins [154], metal ions [155], cells [156], and other types of molecules [157]. Aptamers are widely used in biomarker detection due to their simple synthesis, ease of functionalization, and thermal and chemical stability [131,155]. Additionally, aptamers undergo immediate and reversible conformational changes upon target binding, which allows for the modification of aptamers with immobilized redox reporters on the surface of electrodes [1,130,158,159]. This enhances electron transfer dependent upon target presence. Electrochemical aptamer-based (EAB) sensors allow for continuous measurement of the target due to the reversible recognition [159]. Accordingly, EAB sensors combined with MNs provide an ideal platform for measuring biomarkers in dermal ISF and for drug delivery monitoring [159,160]. However, integrating EAB sensors with MNs for continuous monitoring of molecules in ISF presents several challenges. Specifically, during the process of skin insertion, functionalized aptamers on the MN surface may be lost, and the electrode may suffer damage, both of which could affect signal stability [37,130]. Additionally, the MN surface should be highly biocompatible and satisfy electrochemical requirements such as high sensitivity and resistance to contamination [8].

**Figure 5:**
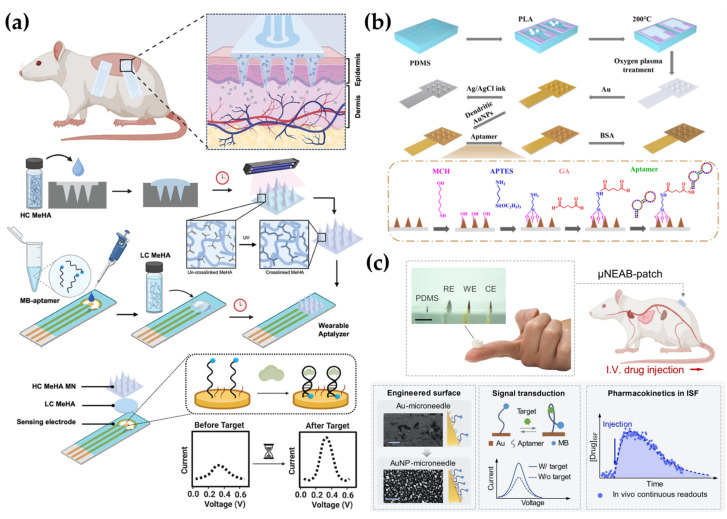
Aptamer-based detection of electrochemical MNs in ISF detection. (**a**) Schematic of the fabrication of the Wearable Aptalyzer and in vivo sensing process with target-specific recognition. Reprinted with permission from [130]. Copyright 2024, Wiley-VCH. (**b**) Schematic illustration of the fabrication of MNs deposited with dendritic AuNPs and their aptamer modification. Reprinted with permission from [131]. Copyright 2024, Elsevier. (**c**) Schematic diagram of the μNEAB sensor and the aptamer-target binding process. Reprinted with permission from [8]. Copyright 2022, AAAS.

To address these challenges, various strategies have been explored to enhance the stability and functionality of EAB-integrated MN sensors for continuous monitoring in ISF. As an example of this approach, Bakhshandeh et al. [130] developed a Wearable Aptalyzer by integrating a MeHA HMN with an EAB sensor to monitor glucose and lactate in real time. The HMN patch swells upon dermal insertion, allowing analytes to diffuse to an electrode functionalized with a methylene blue (MB)-tagged aptamer, thereby minimizing the damage to both the probe and the electrode. Ex vivo tests with aISF showed that the glucose Aptalyzer measured concentrations from 0 to 50 mM with an LOD of 2.4 mM, while the lactate Aptalyzer detected lactate concentrations from 0 to 20 mM with an LOD of 1.04 mM. Long-term in vivo stability was confirmed by monitoring the lactate sensor on rat skin for three days, demonstrating reliable signal generation. Both sensors were used simultaneously in a rat model with a larger skin surface area to detect glucose and lactate in a type 1 diabetic rat, yielding results similar to blood-based measurements (Figure 5a). In a different study, Dervisevic et al. [37] developed a polymeric MN array protected with a polymeric lattice membrane (PL-pMNA) that combines a layer for electrochemical signaling with a protective layer covering the MN to prevent damage. This system was applied for metabolite detection using glucose and hormone biomarker detection using insulin. They prepared the primary master mold using 3D printing and then created a secondary master mold with PDMS. Three types of molds were used: one was used to prepare the Au-coated pMNA for electrochemical measurements, while the other two were used to manufacture PL membranes functioning as protective layers. Using the PL-pMNA/aptamer, they were able to measure glucose in the range of 3 to 21 mM and insulin in the range of 0.2 to 2.0 nM. The sensor showed no significant degradation in performance during insertion into porcine cadaver skin, even after the third application, thus demonstrating the system’s reliability. Yue Jing et al. [131] used dendritic AuNPs electrodeposited onto MNs to expand the surface area for aptamer conjugation and subsequently enhance detection sensitivity. By depositing dendritic AuNPs with a large surface area onto the MN, the conductivity and electrochemical activity of the electrode were enhanced, improving the charge transfer rate. As a result, a signal improvement effect was achieved. Additionally, by modifying the aptamer with an amine group, it was easily conjugated to the AuNPs, resulting in a stable binding. The group detected cortisol in ISF, a hormone known to regulate processes such as glucose levels and carbohydrate metabolism. The detection range for cortisol concentrations was 1 to 1000 nM, with LODs of 0.17 nM and 0.22 nM in phosphate-buffered saline (PBS) and simulated ISF, respectively. The cortisol concentrations measured using the MN sensor were consistent with those obtained through LC/MS measurements, demonstrating the effectiveness of the MN biosensor for cortisol detection (Figure 5b).

Furthermore, biosensor systems combining EAB sensors and MNs have also been widely applied to TDM for drugs with narrow therapeutic windows, such as antibiotics including vancomycin and tobramycin [8]. Monitoring of pharmacokinetics, pharmacodynamics, and toxicology for individual patients can contribute to achieving more efficient individualized drug therapy [161].

Wu and colleagues conducted research to expand the detection range of MN sensors using EAB sensors. They first fabricated MN sensors using biocompatible methyl methacrylate-based resins through 3D printing. The sensors were then placed on the skin of rats and used to continuously monitor levels of the antibiotic tobramycin following intravenous (IV) administration. While the sensor was positioned on the skin, voltage-current characteristics were measured in real-time at a frequency of 80 Hz, which showed changes in current corresponding to the timing of tobramycin IV administration [1]. To minimize exposure to impurities such as Ni and enhance aptamer binding affinity, Lin et al. [8] developed a μNEAB sensor by coating clinically graded, cost-effective needles with AuNPs, which resulted in an improved S/N ratio. The surface of the modified MN was functionalized with an aptamer labeled with MB, enabling the measurement of changes in charge transfer rate upon target drug binding via voltammetry. Using the μNEAB sensor, they successfully detected vancomycin, doxorubicin, thrombin, and tobramycin. Among these, they specifically evaluated the sensor’s performance for tobramycin, confirming that in aISF, the signal drift remained below 10% after 1000 repeated scans and after 15 h of continuous operation. Furthermore, testing in an ex vivo phantom gel setup demonstrated a rapid and stable response to tobramycin in less than one minute. For testing biofouling resistance, the researchers employed two strategies. The first method involved measuring the sensor response in a BSA-spiked buffer solution to mimic the presence of high molecular weight proteins. The second method evaluated its performance in tissue environments. Specifically, they record the response continuously for five hours on rat or porcine skin, revealing no significant changes in signal intensity. Consequently, the response signal for tobramycin before and after the biofouling tests proved to be statistically nonsignificant (Figure 5c). Downs et al. [39] developed MN sensors by incorporating embedded EAB sensors within commercial-off-the-shelf (COTS) stainless-steel MNs. The electrode is protected in stainless steel, allowing it to easily penetrate the skin without causing noticeable damage to the electrode or displacement of its position within the needle after skin insertion. Additionally, when inserted into porcine skin at room temperature, the electrode generated stable signals during repeated and continuous scanning. Measurement of vancomycin in body-temperature PBS using these sensors demonstrated an accuracy within ± 20%, meeting the requirements for clinical monitoring. They detected a noticeable signal loss during measurements at 10 and 25 Hz frequencies on the skin, which corresponded to the typical biofouling-induced drift phase. For correction of the signal, the researchers applied kinetic differential measurements (KDM) and smoothed the voltammograms using the least squares smoothing method. However, the application of such MN-based EAB sensors in humans requires stricter conditions than those for animal models. Friedel et al. [161] demonstrated the feasibility of human application by investigating the fabrication, storage, and in vivo use of a sensor system in which the sensor itself remains external while only an FDA-approved hollow MN is inserted into the body. In these studies, various technological approaches such as electrode design for improved electrochemical performance, advanced immobilization techniques, and biocompatible materials usage are employed to ensure the stable function of aptamers and improve signal sensitivity. These strategies are expected to significantly enhance the in vivo applicability of aptamer-based MN sensors and serve as an important foundation for practical implementation in drug monitoring and precision biomarker analysis.

### 4.4. Antibody-Based Detection

Electrochemical immunosensors detect and quantify the electrical changes that occur when an antigen binds to an antibody [162]. Antibodies have a strong binding affinity for specific antigens that allows them to provide high selectivity and sensitivity, thus effectively distinguishing target molecules even in the presence of biological interference [163,164]. For biomarker detection, these features of high sensitivity at trace concentrations, fast response time, and excellent target specificity are essential [165]. As a result, electrochemical immunosensors can provide more accurate and selective biomarker monitoring than conventional chemical sensors. However, antibodies are prone to denaturation in biological environments, detach from the electrode surface, and interfere with electron transfer [166]. Overcoming these limitations can significantly improve the performance and stability of antibody-based sensors. Various surface engineering strategies have been developed to enhance the stability and performance of electrochemical immunosensors in biological environments.

**Figure 6:**
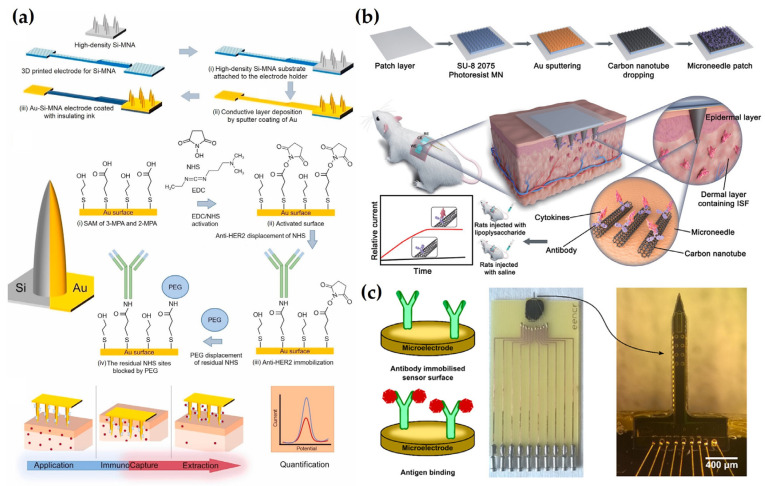
Antibody-based detection in ISF using electrochemical MNs. (**a**) Schematic diagram of Au–Si MN array immunosensor for selective HER2 detection in ISF. Reprinted with permission from [132]. Copyright 2021, Elsevier. (**b**) Pictorial scheme of MN patch for cytokine detection in ISF using CNT-enhanced electrochemical sensing. Reprinted with permission from [133]. Copyright 2023, Wiley-VCH. (**c**) Schematic illustration of a needle-shaped, antibody-functionalized microelectrode array for electrochemical antigen detection. Reprinted with permission from [134]. Copyright 2019, Elsevier.

For example, Dervisevic et al. [132] fabricated an electrochemical immunosensor-based MN sensor for detecting human epidermal growth factor receptor 2 (HER2), a breast cancer biomarker. Anti-HER2 antibodies were covalently immobilized onto high-density silicon MN arrays (Au–Si-MNA) using 1-ethyl-3-[3-dimethylaminopropyl] carbodiimide hydrochloride (EDC) and N-hydroxysuccinimide (NHS) coupling chemistry. To improve the stability and uniformity of the electrode surface, 2-mercaptoethanol was used for backfilling. In addition, a poly(ethylene glycol) bis(amine) (PEG-diamine) layer was introduced to suppress nonspecific protein adsorption and to maintain the structural integrity of the immobilized antibodies. An insulating layer was applied to the electrode surface to minimize signal interference from the skin while preserving electron transfer efficiency. The sensor exhibited stable performance after 20 days of storage and 15 cycles, and maintained high selectivity for HER2 even in the presence of diverse potential interferents. It achieved LODs of 4.8 ng/mL and 25 ng/mL in aISF and a gel-based skin model, respectively, validating its potential for noninvasive monitoring of HER2 in ISF without blood sampling (Figure 6a). Focusing on cytokine storm monitoring, Xu et al. [133] developed a wearable MN patch for early warning and electrochemical detection of inflammatory cytokines interleukin-6 (IL-6), interleukin-1β (IL-1β), and tumor necrosis factor-alpha (TNF-α) in ISF. To enhance signal sensitivity, the electrode was fabricated using CNT-chitosan composites. Anti-cytokine antibodies were directionally immobilized using EDC/NHS coupling chemistry to ensure selectivity and stability, while BSA treatment was employed to suppress nonspecific adsorption and preserve antibody functionality. This surface treatment also contributed to minimizing biofouling and maintaining sensor reliability under physiological conditions. The sensor demonstrated a wide detection range for IL-6 (1–5000 pg/mL) in aISF, with a LOD of 0.54 pg/mL, indicating high sensitivity. It maintained selectivity in the presence of various interferents and stable performance after five days of storage and three days of continuous usage. In a sepsis-induced mouse model, the sensor successfully detected early elevations in cytokine levels, with ISF IL-6 concentrations showing a strong correlation (Pearson r > 0.95) with serum ELISA measurements. This study highlights the feasibility of MN patch platforms for inflammatory biomarker monitoring by combining antibody stability with accurate electrochemical signal output (Figure 6b). In a related effort to monitor inflammatory cytokines, Russell et al. [134] developed an electrochemical MN sensor for IL-6 detection. The sensor is based on a silicon microelectrode array equipped with Au disk electrodes designed to detect electrochemical signal changes associated with antigen-antibody interactions. To prevent antibody desorption and denaturation while ensuring stable signal output, the antibody was covalently immobilized using sulfo-LC-SPDP, and the electrode surface was stabilized by backfilling with 6-mercapto-1-hexanol. Additionally, BSA treatment was applied to reduce nonspecific protein binding and protect antibody activity. The sensor achieved an LOD of 0.54 pg/mL within a detection range of 1 to 5000 pg/mL in aISF. It maintained high selectivity in the presence of interference, with a stable performance after 5 days of storage and three days of continuous utilization. In a sepsis-induced mouse model, the sensor accurately tracked the increment of IL-6 concentration, with ISF measurements displaying a strong correlation with serum ELISA, achieving a Pearson r value exceeding 0.95. This study highlights the possibility of wearable MN sensors for IL-6 monitoring, suggesting their capacity for early sepsis diagnosis and inflammation tracking (Figure 6c). In contrast to enzyme- or aptamer-based strategies, studies using antibody-immobilized electrochemical MN sensors for biomarker monitoring are still relatively limited. This is due to technical challenges in achieving stable antibody immobilization on the electrode surface [167], high production costs of antibodies [168], and difficulties in efficient electrochemical signal transduction [169]. Nevertheless, the studies discussed above demonstrated promising technological approaches to overcome these limitations by improving antibody immobilization strategies and enhancing the electrochemical performance of electrode materials. These advances contribute to increased signal sensitivity and reproducibility, thereby enhancing the capability of antibody-based MN sensors to be used as a practical immunosensing platform for in vivo applications.

### 4.5. Other Detection Methods

With the advancement of electrochemical sensor technology, various new electrochemical MN sensors with different principles and structural designs have been proposed. These alternatives can be an effective solution depending on the specific application or operational requirements. This section provides an overview of the working principles and application cases of these alternative approaches and discusses practical implementation possibilities and future development directions.

**Figure 7:**
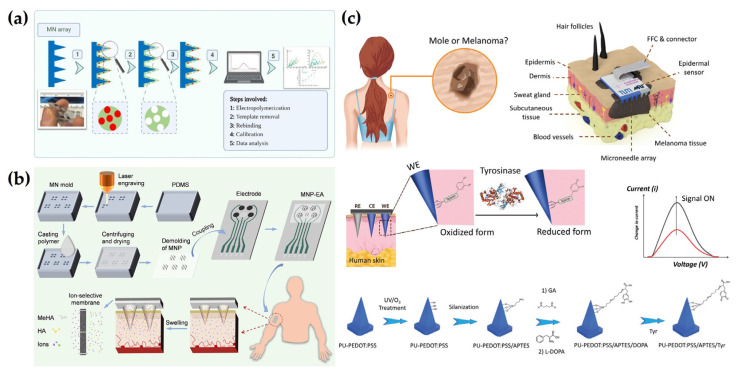
Alternative detection strategies in ISF using electrochemical MNs. (**a**) Scheme of an MIP-functionalized MN sensor enabling selective and label-free IL-6 detection. Reprinted with permission from [135]. Copyright 2021, American Chemical Society. (**b**) Schematic diagram of an electrode-integrated MN patch (MNP-EA) for real-time, label-free detection of Na^+^, K^+^, Ca^2+^, and pH using ion-selective membranes. Reprinted with permission from [170]. Copyright 2023, American Chemical Society. (**c**) Schematic illustration of a tyrosinase-responsive MN sensor patch for melanoma detection via epidermal application and smart probe-based electrochemical sensing. Reprinted with permission from [136]. Copyright 2024, Wiley-VCH.

Among these approaches, Oliveira et al. [135] developed a molecular imprinting polymer (MIP)-based electrochemical MN sensor for IL-6 detection. The sensor was designed to generate an electropolymerized MIP layer based on 3-aminophenylboronic acid (3-APBA) using IL-6 as a template molecule. Following template removal via protease treatment, a metallization process was applied to enhance electrode conductivity and signal detection. In addition, a metallization process was applied to increase the conductivity of the electrode and facilitate the detection of electrochemical signals. In aISF, the sensor demonstrated ultrasensitive performance with a detection range of 1 pg/mL to 10 ng/mL and a LOD of 1.0 pg/mL. Compared to antibody-based immunosensors, the MIP-based platform offers improved stability, reusability, and potential for cost-effective mass production. This study highlights the potential of MIP-integrated MN sensors as a robust alternative for cytokine detection without the need for biological recognition elements such as antibodies or enzymes. Further validation through in vivo and clinical studies is warranted to confirm its applicability in real biological environments (Figure 7a). In another study, Zhu et al. [170] developed an MN-based electrochemical patch for monitoring of sodium (Na^+^), potassium (K^+^), calcium (Ca^2+^), and pH levels in ISF. The sensor utilized hydrophilic MNs composed of MeHA and cross-linked HA to enable efficient ISF extraction through capillary action. Electrodes functionalized with ISMs allowed for the selective detection of target ions. The sensor demonstrated applicability in both animal and human models, enabling continuous and noninvasive monitoring of electrolyte fluctuations during physiological changes such as dehydration. This study highlights the potential of MN platforms for wearable electrolyte sensing in personalized health tracking and disease management (Figure 7b). Poursharifi et al. [136] developed a chemically responsive MN sensor patch incorporating a smart probe for detecting tyrosinase (Tyr), a key biomarker for skin health and melanoma. The sensor utilized an L-3,4-dihydroxyphenylalanine (L-DOPA)-based probe that undergoes oxidation to o-dopaquinone (o-DQ) in the presence of Tyr, generating a quantifiable electrochemical signal. The device demonstrated high selectivity, reusability, and long-term storage stability, maintaining performance after multiple skin insertions and 120 days of storage. In aISF, the sensor achieved a limit of detection (LOD) of 0.06 mg/mL, while in ex vivo human skin models, it successfully differentiated Tyr-rich from Tyr-deficient tissues with stable signal output. These results underscore the potential of smart probe-integrated MN systems for noninvasive enzyme biomarker detection and early-stage melanoma diagnosis (Figure 7c). These approaches complement the limitations of conventional biorecognition element-based sensors and contribute to expanding the applicability of electrochemical MN sensors. Ultimately, strategies that incorporate inherent selectivity through structural design or leverage physicochemical reactions offer practical advantages such as enhanced stability and long-term usability. As a result, electrochemical MN sensors are increasingly regarded as promising tools for real-world clinical diagnostics and disease monitoring applications.

## 5. Smart Electrochemical MN Systems for Advanced Diagnostics

Advances in electrochemical MN wearable sensors have driven significant changes in a variety of fields, including medicine [19], sports [171], and personal healthcare [172]. Sensor technology has evolved into progressively miniaturized and user-friendly wearable forms that enable continuous health monitoring during daily activities while attached to the skin [171,173]. The following studies further demonstrate the expanding potential of MN technology within the realm of wearable sensing applications.

**Figure 8:**
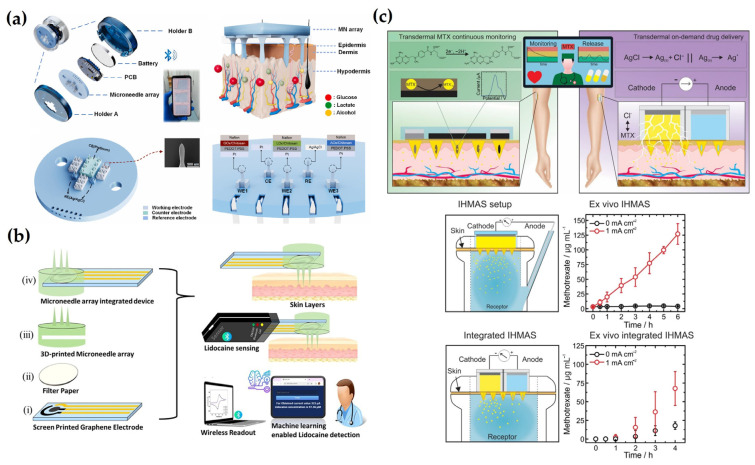
Recent advances in electrochemical MN wearable sensors for personal healthcare. (**a**) Schematic diagram of a wearable MN-based multiplexed sensor system in ISF with Bluetooth signal transmission capabilities. Reprinted with permission from [176]. Copyright 2024, Elsevier. (**b**) Schematic illustration of a wearable MN-integrated electrochemical sensor for lidocaine detection featuring wireless signal transmission and machine learning-enabled data analysis. Reprinted with permission from [179]. Copyright 2024, Elsevier. (**c**) Graphical scheme of a wearable MN-based platform for MTX monitoring and on-demand iontophoretic drug delivery demonstrating sensing and controlled release capabilities in in vitro models. Reprinted with permission from [182]. Copyright 2023, American Chemical Society.

For example, Yang et al. [174] presented a miniaturized, high-precision, and fully integrated wearable electrochemical MN sensor that works with a custom smartphone application for wireless glucose monitoring in ISF. The system consists of a disposable MN sensor and a reusable electronic module that continuously collects glucose signals from ISF and transmits them to the smartphone application. This setup facilitated advanced data visualization and analysis for health monitoring. While single-analyte-based systems are effective in monitoring specific biomarkers, their ability to provide comprehensive physiological information remains limited. As a result, multiplex sensor technologies that can simultaneously detect multiple biomarkers have attracted increasing attention. Such platforms can improve diagnostic accuracy and enable a more accurate assessment of overall health status [175]. Reflecting this potential, Zhong et al. [176] developed a multifunctional MN patch that can simultaneously detect multiple physiological indicators. Each MN was functionalized with Pt, conductive polymer, and enzyme layers designed to detect fitness-related biomarkers, including glucose, lactate, and alcohol. A miniature signal processing circuit and a wireless communication module were then integrated for real-time data transmission. The electrochemical response of the sensor showed a consistent linear correlation with the change in biomarker concentration, confirming high sensitivity and accuracy. The collected data were then transmitted to a smartphone via Bluetooth and uploaded to a cloud-based platform for further health analysis. This study highlights the potential of smart wearable devices to enhance sports and fitness monitoring and contribute to the development of next-generation wearable health technologies (Figure 8a). Additionally, Gao et al. [177] developed a biosensor integrating a multichannel portable electrochemical analyzer and MN electrode arrays for the simultaneous detection of glucose, UA, and cholesterol. The MN electrode arrays were fabricated using magnetorheological drawing lithography and functionalized with specific enzymes for each biomarker. The sensor exhibited excellent detection performance with LOD limits of 260 μM, 4 μM, and 440 μM for glucose, UA, and cholesterol, respectively, and a fast response time of about 4 s. The integrated electrochemical analyzer can accurately detect multiple biomarkers simultaneously, providing a convenient solution for effective monitoring of metabolites.

These technological advancements are progressively transforming how we analyze and interpret collected physiological data beyond the simultaneous detection of multiple biomarkers. In particular, the integration of AI can significantly enhance the capabilities of wearable MN sensors, transitioning them from static measurements to dynamic biosensing systems that can predict physiological responses and health status changes. AI-based systems can detect subtle changes in biomarker levels and predict disease progression, offering personalized feedback to users. This convergence of technologies represents a groundbreaking leap forward in diagnostic innovation and paves the way for more intelligent, sensitive, and personalized health monitoring solutions [178]. Therefore, future research will be expected to focus on developing advanced algorithms and machine learning models for continuous data processing to enable early detection and prediction of abnormal health patterns based on the collected biosensing data. Kadian et al. [179] designed a wireless, MN array-integrated, screen-printed electrode-based POC electrochemical device capable of machine learning-assisted detection of lidocaine. The system collected ISF from the bottom of the MN and then measured the electrochemical signal using graphene-modified screen-printed carbon electrodes. Accordingly, a machine learning model was trained on experimental sensing data to predict lidocaine concentrations, and the model was deployed on a web-based application for digital visualization of the results. The model’s high predictive accuracy and user-friendly digital interface facilitate field usability and accessibility. As a result, the sensor exhibited a linear current response in the range of 1 to 120 μM with an LOD of 0.13 μM (Figure 8b).

Real-time data analytics and predictive technologies are opening new possibilities for integrated systems that go beyond diagnostics to include therapeutic capabilities. Theragnostic MN systems that combine monitoring and drug delivery capabilities can detect biomarker fluctuations in real time and dynamically adjust drug dosages accordingly [180]. Such systems are suitable for personalized therapy, allowing self-monitoring and remote management, which can significantly improve the treatment and long-term management of chronic diseases [181]. One application of this concept is the HMN patch developed by Parrilla et al. [182], which enables monitoring and on-demand delivery of methotrexate (MTX) via iontophoresis. The sensor has enhanced MTX adsorption on the electrode surface using graphite paste and chitosan-based coating. When voltage is applied, MTX is oxidized, thus generating a quantifiable electrochemical signal. In aISF, the sensor showed linearity over the concentration range of 25 to 400 μM, with an LOD of 10 μM. The sensor performed reliably in a porcine skin model and maintained high reusability with less than 5% signal change after 5 consecutive days of use. During drug delivery experiments, iontophoresis increased MTX permeability by 26-fold, as evidenced by an increase in drug delivery from 4.5 μg/mL (without iontophoresis) to 118.6 μg/mL in Franz diffusion cell experiments. This study presents a closed-loop therapeutic concept that monitors patient biomarkers and automatically regulates drug delivery, demonstrating the potential of such systems for smart and personalized medicine (Figure 8c).

This research direction expands the functional capabilities of electrochemical MN wearable sensors and maximizes their utility in real-world settings, providing a new paradigm for personalized healthcare and preventive medicine [183]. These sensors can facilitate a proactive approach in healthcare by allowing users to continuously monitor their health status and immediately take action when needed. In particular, smart MN platforms enable simultaneous monitoring of multiple biomarkers, automated signal interpretation with predictive insight, and therapeutic feedback—all within a skin-conformal, minimally invasive format—thereby redefining diagnostic precision and clinical responsiveness in modern healthcare. This ultimately contributes to improving the quality of life by enhancing individuals’ health awareness and self-management abilities, further representing a significant technological advancement [184].

## 6. Conclusions and Future Perspectives

This review examines the technologies and methods used to monitor various biomarkers and drugs in the ISF of the skin in real time using electrochemical MN sensors. First, functional nanomaterials such as conductive polymers, metals, and 2D materials that can improve electrochemical sensitivity while minimizing skin damage are introduced. Then, various microfabrication techniques, such as lithography and 3D printing, were compared and analyzed. These precision manufacturing processes improve the mechanical stability and insertion precision of the MNs while enabling sensor miniaturization and multifunctional integration. In addition, various electrochemical sensing mechanisms have been summarized, ranging from direct electrode-based sensing methods to those based on biorecognition elements such as enzymes, aptamers, and antibodies. As each receptor type exhibits different selectivity, stability, and sensitivity depending on the target analyte and analytical conditions, the strategic selection of receptors is critical to ensuring diagnostic accuracy and reliability. Recently, wearable platforms based on electrochemical MN sensors have attracted attention in various fields such as clinical medicine, sports, and personal health monitoring. Their ability to adhere to the skin for extended periods and continuously measure biosignals in real time represents a significant technological advancement. In particular, the integration of sensor array technology for the simultaneous detection of multiple biomarkers, machine learning-based data analysis, and theragnostic approaches has shown the potential to provide higher information density and predictive capability compared to conventional diagnostic systems. These technological advances are extending beyond the research stage and increasingly demonstrating feasibility for implementation in clinical settings.

Currently, several companies are actively developing electrochemical MN sensors with the goal of commercialization. Biolinq has developed a coin-sized wearable patch capable of measuring biomarkers such as glucose, lactate, and ketones from ISF, ensuring high precision and durability. Sava Technologies has developed an electrochemical sensor utilizing multiple MNs approximately 1 mm in length and is preparing for clinical trials targeting diabetic patients in the United Kingdom. The platform also demonstrates expandability for monitoring additional biomarkers, including ketones and urea. Nutromics has introduced a therapeutic drug monitoring system for vancomycin using an aptamer-based electrochemical MN sensor, with clinical trials currently underway in healthy adults. The company is focusing on product development and scaling up manufacturing, aiming for commercialization by 2027. These examples illustrate the growing maturity of electrochemical MN sensor technologies and their increasing readiness for practical implementation in real-world healthcare settings. Despite these developments, there are still several important challenges to the commercialization of electrochemical MN sensors. First, biomarker concentrations in ISF exhibit significant variability among individuals and are influenced by physiological and environmental factors such as sweat, temperature, and physical activity [185]. Therefore, the development of sophisticated algorithms and signal processing techniques capable of real-time data calibration is essential [186]. Second, in order for MN sensors to be competitive with the existing system in terms of production cost, it is essential to establish process technologies capable of mass production, and for this, research on automation and cost-effective manufacturing technology requires further advancement [187,188].

Ultimately, electrochemical MN sensors are expected to play an important role in personalized medicine and digital healthcare industries based on the advantages of minimally invasive, high-sensitivity analysis, and continuous monitoring. The convergence of various sensing mechanisms, precision manufacturing technologies, and biocompatible materials discussed in this review suggests that these technologies serve as the basis for expansion not only into the clinical environment but also to everyday health care. Achieving this vision will require interdisciplinary collaboration and ongoing technological innovation worldwide.

## Figures and Tables

**Table 1:** Comparison of fabrication methods for electrochemical MN sensors.

Method	Resolution	Scalability	Material Compatibility	Reference
Photolithography	3 μm	Pilot scale	Si wafer	[91]
			PEGDA	[92]
Casting and molding	3 μm	Industrial scale	SWCNT	[93]
			liquid PI	[94]
3D printing	2 μm	Pilot scale	Bio resin	[95]
			Clear light-sensitive resin	[96]
Laser cutting	1.06 μm	Pilot scale	PI film	[97]
			Stainless steel	[98]

**Table 2:** Comparative summary of receptor types used in electrochemical MN sensors.

Receptor Type	Target Analytes	Linear Range	Advantages	Disadvantages	Lab to Commercial Translation	Reference
Direct redox	DA, EP, NEP	0.5–100 µM, 0.5–75 µM, 0.5–75 µM	Eco-friendly, low-cost, and highly reproducible electrode modification process	Difficulty in individual quantification of DA, EP, and NEP	Requires future work for wireless integration and in vivo deployment	[125]
	Serotonin (5-HT)	0–95 μM	Simple fabrication process	Difficulty in ensuring manufacturing consistency	Wearable potential demonstrated, but no in vivo validation yet	[74]
	UA	0–500 μM	Anti-biofouling and reusable	Limited scalability and insufficient process automation	Despite limited commercialization, precision manufacturing via 3D printing and related techniques proposed	[126]
Enzyme	Glucose	0–20 mM	Low cost and good portability	Reduced sensitivity at high glucose and limited practicality due to electrode integration	Low-cost and validated in lab, yet electrode integration and fabrication hurdles limit clinical translation.	[127]
	Cholesterol	1–15 mM (in aISF)	High selectivity and long-term stability	Stabilizing materials required to maintain enzyme activity and prevent leaching	Lab performance validated, future wearable integration suggested	[128]
	β-lactam antibiotic	approximately 10–800 μM	High specificity, stability after sterilization, and good storage stability	Sensitivity reduction due to initial enzyme leaching	In vivo testing demonstrates feasibility, but further sensitivity optimization needed for clinical deployment	[129]
Aptamer	Glucose, Lactate	0–50 mM, 0–20 mM	High sensitivity and specificity in real skin environments	Multiplex detection via two sensors, and sensor fabrication complexity	Testing on human skin or subjects for in vivo monitoring	[130]
	Cortisol	1–1000 nM	High performance, stability, repeatability, and immunity to interference	Multi-step sensor fabrication	Long-term in vivo testing for continuous monitoring	[131]
	Vancomycin	6–42 μM (clinical window)	Biocompatible, sterilizable and stable	Small redox currents due to small working electrode surface area	Translation into in vivo and enhancement of surface area for accuracy and multiplexing	[39]
Antibody	HER2	10–250 ng/mL (in aISF)	Dual-function platform with high sensitivity and specificity	Structural instability of the SAM and high fabrication cost	Lab performance validated and in vivo application suggested	[132]
	IL-6, IL-1β, TNF-α	1–5000 pg/mL	High sensitivity, specificity, and stable performance	High cost and limited long-term storage	Difficult to scale up due to antibody immobilization and in vivo application limitations	[133]
	IL-6	0–60 pg/mL	High specificity without the need for complex surface modification	Structural instability of the SAM and high fabrication cost	Lab performance validated and in vivo application suggested	[134]
MIP	IL-6	1 pg/mL–10 ng/mL	High reusability and low production cost	Lack of binding site precision	Scalable, low-cost platform suitable for POC, but lacks in vivo validation and multiplex capacity	[135]
Chemo-responsive probe	Tyr	0.3–0.7 mg/mL	Reusability enabled via probe regeneration by CV	Manual fabrication limits scalability	Lab performance validated, but not yet ready for mass production	[136]

## Data Availability

Not applicable.
